# Understanding Stability and Change in Perceived Social Support in Parents of Autistic Children and Adolescents

**DOI:** 10.3389/fresc.2021.679974

**Published:** 2021-06-02

**Authors:** Jonathan A. Weiss, Suzanne Robinson, Rebecca Pillai Riddell, David Flora

**Affiliations:** ^1^Developmental Disabilities and Mental Health Laboratory, Department of Psychology, York University, Toronto, ON, Canada; ^2^Roncesvalles Psychology Clinic, Toronto, ON, Canada; ^3^Opportunities to Understanding Childhood Hurt Laboratory, Department of Psychology, York University, Toronto, ON, Canada; ^4^Quantitative Methods, Department of Psychology, York University, Toronto, ON, Canada

**Keywords:** social support, autism, parent stress, behavior problems, longitudinal design

## Abstract

Parents of children with autism often have their own support needs. Informal social support can be an important component of managing parenting-related stressors. We know very little about the factors that lead to higher levels of perceived social support or the potential reciprocal relationship social support has with other factors in parents of children with autism. The current longitudinal study examined the reciprocal relations of perceived social support and parent stress and child behavior problems across a 1-year period, using three time points. There was remarkable stability in variables over time. Baseline perceived social support significantly predicted changes in child behavior and parent stress at the 6-month time point, but neither of those variables significantly predicted social support. This study adds to our understanding of social support and clarifies how perceived social support relates to other factors longitudinally.

## Introduction

The benefits of social support are well-documented for parents of individuals with autism [e.g., (1–7)]. Cohen et al. (8) state that social support is “the social resources that persons perceive to be available or that are actually provided to them by non-professionals in the context of both formal support groups and informal helping relationships” (p. 4). *Perceived social support* is the belief that support is adequate or available if needed, reflecting how supported a person feels rather than specific or concrete supports experienced (9). It appears that perceived support can help with a person's well-being regardless of the stressors experienced (10) and may be particularly important within the context of chronic and acute negative life events (11).

Perceived support is consistently linked to well-being in parents, including lower levels of stress (12), depressive symptoms (13), distress (14), and increased self-confidence (15). This pattern is also found in parents of people with autism as well (2), with perceived support being associated with lower mental health problems and greater life satisfaction and general well-being, in both mothers and fathers (4, 16–19). One of the most commonly studied correlates of perceived support is parents' stress (20), broadly defined as the distress, discomfort, or arousal experienced in response to perceived demands.

Child behavior problems is a particularly relevant variable to consider in reference to perceived support for parents of people with autism. Individuals with autism may struggle with high levels of irritability, emotion regulation problems, aggression, or self-injurious behaviors, which regularly require parent support (21). For example, in a study of 1,380 parents of children and adolescents with autism, nearly 70% reported that their child had demonstrated aggression toward caregiver, and half toward non-caregivers (22). There is some research to suggest that caregivers may struggle to mobilize support or be more reluctant to seek support when children with autism show more difficult behaviors. From interviews with 46 parents of children with autism, Ryan (23) described parent reluctance to enter public places and struggles to find social acceptance. Without obvious outward signs of their child's disability, parents often perceived judgement from the community when their child acted out or pushed societal norms. Similarly, Gray's (24) qualitative study involving 33 Australian parents of school-aged children (4–19 years) with autism found that parents withdrew from their social networks in response to perceived stigma and the stressful nature of public encounters. Many parents report heightened feelings of isolation when their child had aggressive or disruptive behavior, suggesting a potential link between child behavior and perceived availability of support. A decade later, Gray (25) interviewed 28 of these families again to examine how coping changes over time. Parents reportedly felt more comfortable engaging in social activities in the community because they perceived their child's behaviors to have improved, but parents also had grown accustomed to the longstanding social restrictions that existed for their families. Cross-section surveys confirm this negative correlation between child behavior problems and social support, found in studies of very young children to late adolescence (3, 18, 26).

While there is substantial cross-sectional information to support the idea of associations, we know little about the directionality of perceived social support. To date, no study has examined stress as a determinant of perceived social support longitudinally for parents of individuals with autism, or considered a bidirectional relationship between stress or child behavior problems and perceived social support. While it is possible that changes in stressors or child functioning leads to changes in perceived support, it is also possible that greater support leads to improved perceptions of stressors and stress-responses. More broadly, there is evidence that these variables show a degree of stability over short periods of time (27–30) and it is an empirical question as to the degree of change and stability that is witnessed in community samples of stress, perceived support, and child behavior problems. Using online survey data collected from 249 parents of school-aged children with autism, the current study assessed the relationships between perceived social support and parent perceived stress and child behavior problems across three time points, within a 1-year period. While we expected stability in terms of child behavior problems, we also considered that their presence would lead to changes in perceived social support over time, and that changes in support would lead to changes in behavior problems. Similarly, though we would see stability in parent stress levels, above this we expected strong relationships with support over time.

## Methods

### Participants

Baseline data were available for 249 parents who sufficiently completed an online survey (i.e., at least 75% of survey items) and met all eligibility criteria (described below). At time 2 (6 months after baseline), 194 participants responded. At time 3 (12 months after baseline), there were 180 participants (17 of these participants did not respond at time 2). The study had 163 participants complete all three surveys. The 163 participants who sufficiently completed all three time points were compared to the 86 parents who did not. The two groups did not significantly differ on the main study variables or on family and child characteristics including parent education, household income, child age, child autism symptoms, and child adaptive skills (all *p* > 0.05).

As shown in Table 1, parent age ranged from 27 to 62 years (*M* = 43.98, *SD* = 6.2, Median = 44). Participants were primarily mothers (95.6%) and currently married/common law (83.1%). Most parents (81.9%) had graduated college or university. Parents were from suburban (39.9%), urban (39.1%), rural (16.5%), and remote (4.4%) settings across Canada. The children with autism ranged in age from 4 to 18 years (*M* = 11.47, *SD* = 3.95, Median = 11) and most were male (83.1%). Additional child diagnoses from a physician, as reported by parents, included intellectual disability (42.4%), learning disability (37.8%), attention deficit disorder or attention deficit hyperactivity disorder (38.4%), anxiety or depression (37.1%), and behavior or conduct problems (29.0%). Nearly half (45.7%) had at least one chronic health condition, including epilepsy, cerebral palsy, or asthma.

**Table 1:** Parent, household, and child characteristics.

	**N (%) or M (SD)**
*Parent/household variables*	
Age (*n* = 233)	43.98 (6.21) Range: 27–64
Gender	
Female	238 (95.6)
Male	10 (4.0)
Transgender	1 (0.4)
Relationship status (*n* = 248)	
Married/common law	210 (83.1)
Single (never married)	10 (4.0)
Separated/divorced	31 (12.5)
Widowed	1 (0.4)
Education level (*n* = 248)	
High school or less	23 (9.2)
Partial college (at least 1 year)	22 (8.9)
College diploma/university undergraduate degree	150 (60.5)
Graduate degree	53 (21.4)
Annual household income after taxes (*n* = 244)	
$45,000 or less	57 (23.4)
$45,000–95,000	105 (43.0)
$95,000 or more	82 (33.6)
Geographical Location (*n* = 248)	
Suburban area	99 (39.9)
Urban area	97 (39.1)
Rural	41 (16.5)
Remote	11 (4.4)
*Child variables*	
Age	11.47 (3.95) Range: 4–18
Gender	
Female	41 (16.5)
Male	207 (83.1)
Transgender	1 (0.4)
Born outside of Canada	12 (4.8)
Activities of daily living skills (W-ADL)	16.69 (7.11) Range: 0–33
Autism Symptoms (SCQ)	22.17 (6.34) Range: 11–38

*N = 249*.

### Measures

#### Demographics

Parents reported their own age, gender, marital status, and income as well as their child's age, gender, and diagnoses.

#### Autism Symptoms

The Social Communication Questionnaire—Lifetime (SCQ) (31) was used to assess autism symptom severity. The SCQ is an autism symptom screener assessing social and communication behaviors and consists of 40 yes-or-no items. Higher total scores indicate greater autism symptom severity. The SCQ has shown strong internal consistency, as well as good discriminant validity for distinguishing between children with autism and those without (32). In the current study, baseline scores had adequate internal consistency (coefficient α = 0.82).

#### Child Adaptive Behavior

Adaptive behavior was measured as a control variable, using the Waisman Activities of Daily Living Scale (W-ADL) (33). This is a 17-item measure of an individual's independence in performing daily activities (e.g., dressing and undressing or drinking from a cup). Item responses are given using a three-point Likert-type scale, with 0 = *Does not do at all* and 2 = *Independent or does on own*. Total scores range from 0 to 34. The WADL has been used with parents of children with intellectual disabilities [e.g., (34)] and with adolescents and adults with autism and no intellectual disability (35). Maenner et al. (33) report good internal consistency and strong validity, as the scale is highly correlated with other measures of adaptive functioning. In the current study, baseline scores had good internal consistency (coefficient α = 0.92).

#### Child Behavior Problems

Child behavior problems were assessed using the Strengths and Difficulties Questionnaire [SDQ; (36)]. The 25 items assess prosocial behavior, peer relationship problems, conduct problems, hyperactivity, and emotional symptoms. Each item is scored using a 3-point scale (*not true, somewhat*, and *certainly true*) and a total difficulties score is calculated by summing the four problem behavior subscales. Example items include “generally liked by other children,” “easily distracted, concentration wanders,” and “often loses temper.” The scale is meant to serve as a brief behavioral screener and is often used in research involving parents of children with developmental disabilities or autism [e.g., (37, 38)]. In the present study, prosocial behavior and peer subscales were not used because they represent areas of functioning represented in the diagnostic criteria for autism, consistent with other studies [e.g., (21)]. The SDQ has shown good internal consistency, test-retest reliability, and validity for parents of typically developing children (39) and internal consistency has been high in a sample of parents of children with autism (0.97) (38). For the current study, coefficient α = 0.78 for baseline total difficulties (sum of conduct problems, hyperactivity and emotional symptoms).

#### Parent Stress

The Stress subscale from the Depression Anxiety and Stress Scale (DASS-42) (40) is a 14-item scale assessing global perceptions of stress. The stress subscale measures the extent to which individuals had difficulty relaxing, feelings of nervousness, agitation, intolerance, impatience, or irritability in the last week. Item responses are given on a four-point Likert-type scale from 0 (*did not apply to me at all*) to 3 (*applied to me very much or most of the time*), where higher scores suggest more perceived stress. Example items include “I found it difficult to tolerate interruptions to what I was doing” and “I was in a state of nervous tension.” The scale has shown acceptable reliability for parents of children with developmental disabilities or autism [e.g., (41)], with coefficient α of 0.85 in a similar study sample (42). Good validity has been demonstrated with a sample of adult psychiatric patients (43) and a non-clinical sample (44). In the current study, baseline scores had good internal consistency (coefficient α = 0.94).

#### Perceived Social Support

Perceived social support was measured with the Social Provisions Scale (45). The scale provides a summary score of global perceived availability of social support. The 24 items are scored using a four-point Likert-type scale ranging from *strongly disagree* to *strongly agree*, with higher scores suggesting greater perceptions of support. Example items include “I feel part of a group of people who share my attitudes and beliefs,” “there are people I can count on in an emergency,” and “there is someone I could talk to about important decisions in my life.” The scale had excellent internal consistency in a large-scale study of its psychometric properties (coefficient α = 0.92) and good convergent and divergent validity (45). The scale has also shown good reliability in studies involving parents of children with behavior difficulties (46) and autism (47). In the current study, baseline scores had good internal consistency, α = 0.94.

### Procedure

Following approval from University's Research Ethics Board, parents of individuals with autism were recruited through postings on the Canadian autism websites, community organizations, and through an ongoing research database available through the primary researcher's lab. A link to the online consent form and survey was provided and parents were invited to contact the researcher by email or phone to request a paper survey. After parents completed the initial survey, they were invited to complete follow-up surveys 6 and 12 months later.

To be eligible for this study, participants were required to have a school-aged child (between 4 and 18 years of age) with a confirmed diagnosis of an Autism Spectrum Disorder and be able to complete the survey in English. Autism diagnosis was confirmed in two ways. First, the parent confirmed that a professional with the capacity to diagnose provided the child with an autism-related diagnosis (selecting one of the of the following: psychologist, psychiatrist, developmental pediatrician, general pediatrician, family doctor, nurse practitioner, multidisciplinary or developmental team, genetic testing, neurologist) and provided the date of diagnosis. Second, the parent-reported score on the Social Communication Questionnaire—Lifetime (SCQ) (31) was above a pre-specified cut-off score of 11, indicating a possible autism diagnosis (48).

### Data Analysis Plan

Two separate autoregressive cross-lagged path models were calculated, allowing for individual examination of stress and child behavior problems with perceived social support across the three time points. This type of statistical model is used to examine transactional relationships between variables and has recently been used in the field of autism research [e.g., (35, 38, 49, 50)]. The model allows for examination of the directionality of effects between two variables measured over time while also considering auto-regression, which is variable stability across time points. Model fit was assessed using a series of common fit statistics such as comparative fit index (CFI), root mean square error of approximation (RMSEA), and Tucker-Lewis index (TLI). The individual parameter estimates pertaining to the cross-lagged effects were subsequently interpreted. Robust maximum likelihood estimation (MLR) (51) was used to account for the possibility of multivariate non-normality and for its effectiveness in dealing with missing data. Demographic variables that showed a significant association with model variables at the bivariate level were included as control variables, to account for many additional stressors that could inadvertently influence the presence of stress, child behavior problems and support, ultimately representing more conservative findings.

## Results

As shown in Table 2, the means and within-variable correlations indicated considerable stability. Further, at Time 1, perceived support was correlated with child adaptive behavior level (*r* = 0.22, *p* < 0.001), child autism symptom severity (*r* = −0.18, *p* = 0.003), the presence of at least one chronic health condition [*t*_(244)_ = 2.0, *p* = 0.04], parent education level (*r* = 0.27, *p* < 0.001), and household income level (*r* = 0.22, *p* = 0.001). Child behavior problems was associated with child autism symptom severity (*r* = 0.15, *p* = 0.02), and parent education level (*r* = −0.18, *p* = 0.005). Stress was associated with child adaptive behavior (*r* = −0.19, *p* = 0.003), child autism symptom severity (*r* = 0.15, *p* = 0.02), and parent education level (*r* = −0.16, *p* = 0.01). Higher perceived social support was significantly related to lower levels of parent stress (*r* = −0.44 *p* < 0.001) and child behavior problems (*r* = −0.17, *p* = 0.01). Given this pattern, household income, parent education, presence of child chronic health conditions, child adaptive behavior level, and child autism symptom severity were entered as control variables in both path models.

**Table 2:** Descriptive and within-variable correlations of main study variables across time points.

	**Baseline**	**6 months**	**12 months**	**T1-T2**	**T2-T3**	**T1-T3**
	**M (SD)**	**M (SD)**	**M (SD)**	***r***	***r***	***r***
Perceived social support (SPS)	75.06 (11.85)	76.33 (11.46)	74.46 (12.29)	0.77^*^	0.78^*^	0.80^*^
Stress (DASS)	15.46 (8.91)	15.75 (9.43)	14.55 (8.90)	0.60^*^	0.61^*^	0.65^*^
Child behavior (SDQ)	12.86 (5.09)	13.55 (4.72)	13.17 (4.90)	0.65^*^	0.78^*^	0.62^*^

* *p* < 0.05.

### Is There a Reciprocal Relation Between Perceived Social Support and Child Behavior Problems, While Controlling for Continuity Over Time for Both Variables?

**Figure 1:**
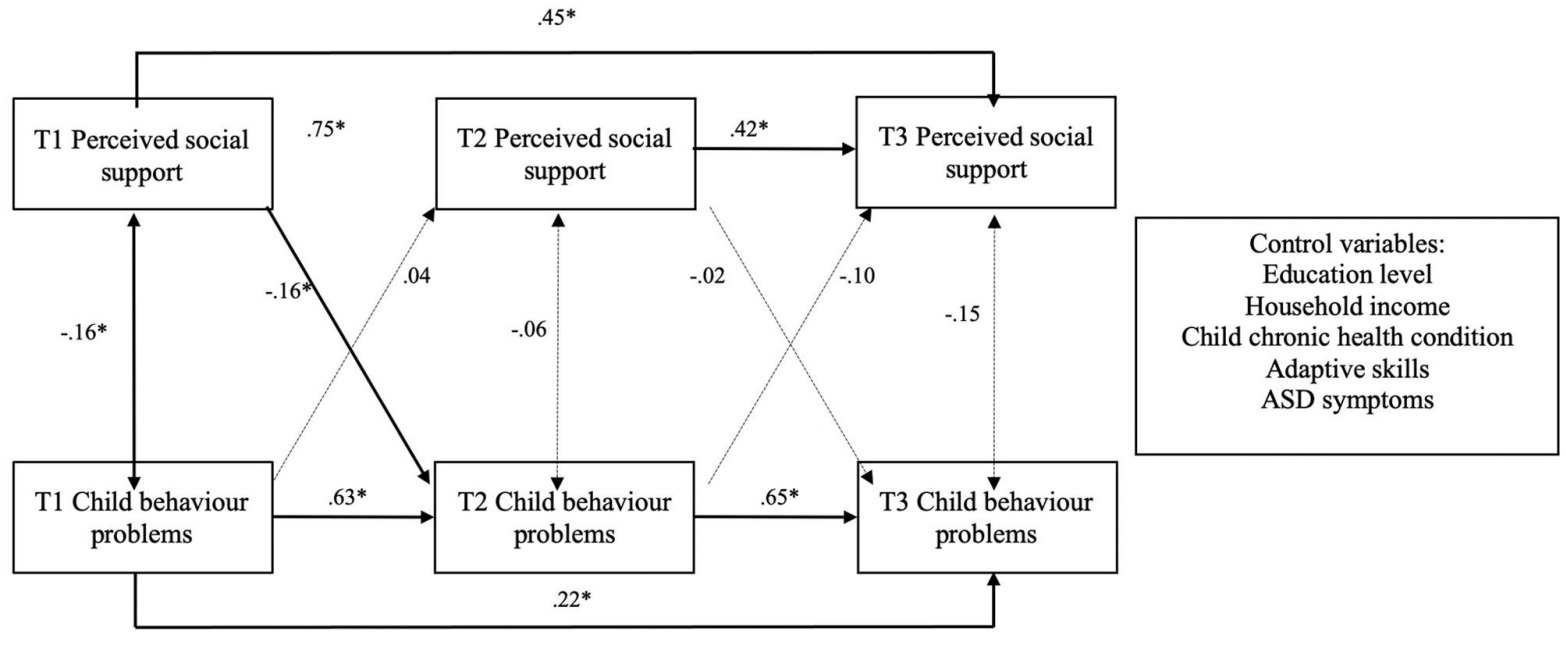
Standardized coefficients of the relationships between perceived social support and child behavior problems across three time points. T1 = baseline; T2 = 6 months; T3 = 12 months; Dotted lines represent non-significant associations; *p < 0.05.

Initial model fit for this model was poor (CFI = 0.86; TLI = 0.56; RMSEA = 0.17; SRM *r* = 0.09). Residual correlations showed strong autoregressive relationships between variables at Time 1 and Time 3, and modification indices suggested that adding direct paths between T1 and T3 would substantially improve the model fit. The adjusted model fit the data well (CFI = 1.0; TLI = 1.0; RMSEA = 0.00; SRM *r* = 0.01). See Table 3 for unstandardized estimates and Figure 1 for the corresponding path diagram with standardized parameter estimates.

**Table 3:** Unstandardized estimates of the relationships between perceived social support and child behavior problems.

	**Estimate (SE)**	***p***
*SS 6 months*		
SS baseline	0.72 (0.05)	<0.001
Behavior baseline	0.08 (0.10)	0.44
Education	0.40 (0.68)	0.55
Household income	−0.07 (0.19)	0.70
Child health condition	−0.82 (1.04)	0.43
Autism symptoms	−0.18 (0.08)	0.02
Adaptive skills	0.10 (0.08)	0.21
Behavior *6 months*		
Behavior baseline	0.58 (0.06)	<0.001
SS baseline	−0.06 (0.02)	0.02
Education	0.04 (0.30)	0.97
Household income	−0.03 (0.09)	0.71
Child health condition	0.19 (0.53)	0.67
Autism symptoms	−0.01 (0.04)	0.95
Adaptive skills	−0.01 (0.04)	0.64
*SS 12 months*		
SS 6 months	0.43 (0.07)	<0.001
SS baseline	0.46 (0.07)	<0.001
Behavior 6 months	−0.24 (0.12)	0.05
Behavior *12 months*		
Behavior 6 months	0.70 (0.07)	<0.001
Behavior baseline	0.22 (0.06)	<0.001
SS 6 months	−0.01 (0.02)	0.72

*SS = perceived social support*.

There were significant autoregressive effects for both perceived social support and child behavior problems, indicating that the prior levels of either variable were strongly related to the same variable's subsequent levels. Specifically, baseline to 6-month social support (*b* = 0.72, *p* < 0.001), 6- to 12-month social support (*b* = 0.43, *p* < 0.001), baseline to 6-month child behavior (*b* = 0.58, *p* < 0.001), and 6- to 12-month behavior (*b* = 0.70, *p* < 0.001) were all significant autoregressive effects. Cross-lagged effects showed baseline social support significantly predicted child behavior problems at 6 months (*b* = −0.06, *p* = 0.02), but baseline behavior did not significantly predict 6-month social support. There were no significant cross-lagged paths from 6 to 12 months.

### Is There a Reciprocal Relation Between Perceived Social Support and Parent Stress, While Controlling for Continuity Over Time for Both Variables?

**Figure 2:**
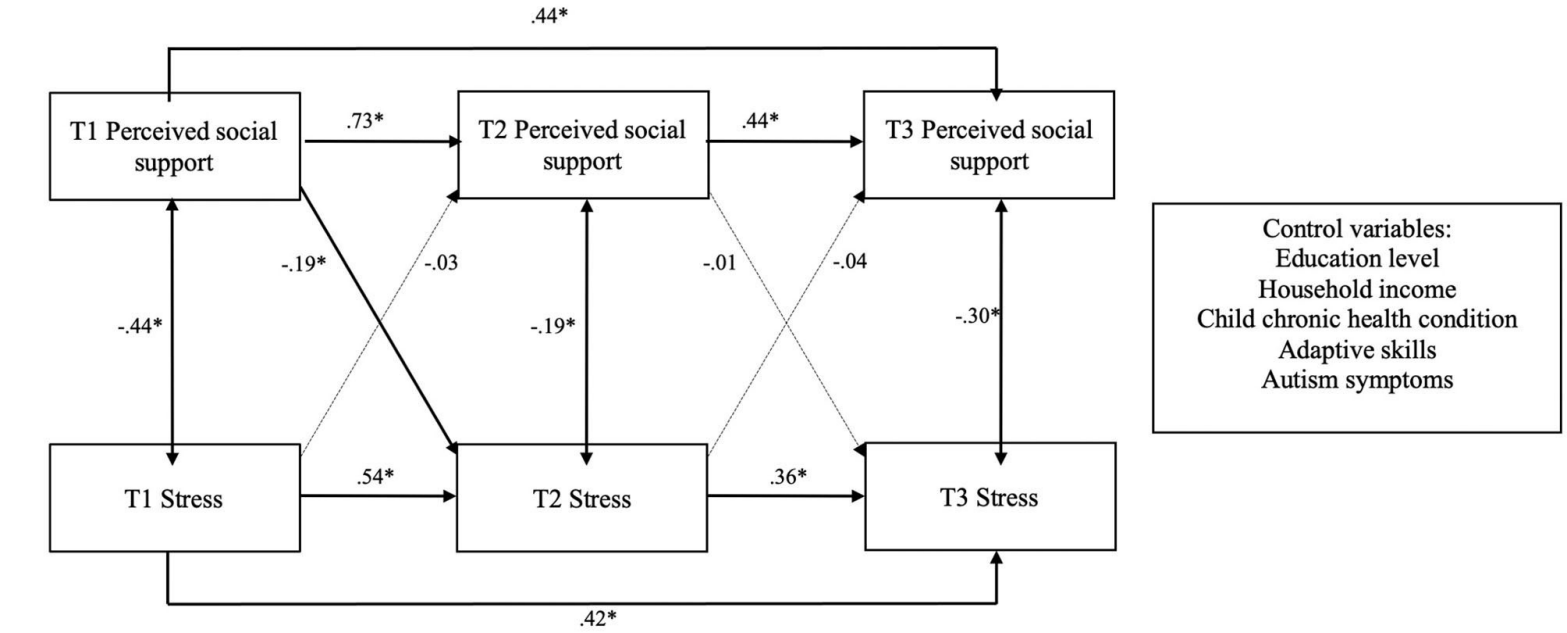
Standardized coefficients of the relationships between perceived social support and stress across three time points. T1 = baseline; T2 = 6 months; T3 = 12 months; Dotted lines represent non-significant associations; *p < 0.05.

The initial planned model with perceived social support and parent stress had an inadequate fit to the data (CFI = 0.84; TLI = 0.70; RMSEA = 0.14; SRM *r* = 0.10). Based on residual correlations and modification indices, direct paths from social support at time 1 to time 3 and from stress at time 1 to time 3 were added to the model. This modification improved model fit such that the adjusted model fit the data well (CFI = 1.0; TLI = 0.99; RMSEA = 0.03; SRM *r* = 0.02). Unstandardized results for this model are reported in Table 4. As shown in Figure 2, both perceived social support and stress were stable over time. Specifically, autoregressive coefficients from baseline to 6-month social support (*b* = 0.71, *p* < 0.001), 6- to 12-month support (*b* = 0.47, *p* < 0.001), baseline to 6-month stress (*b* = 0.57, *p* < 0.001), and 6- to 12-month stress (*b* = 0.33, *p* < 0.001) were all significant.

**Table 4:** Unstandardized estimates of the relationships between perceived social support and stress.

	**Estimate (SE)**	***p***
*SS 6 months*		
SS baseline	0.71 (0.05)	<0.001
Stress baseline	−0.04 (0.06)	0.43
Education	0.29 (0.65)	0.66
Household income	−0.04 (0.19)	0.81
Child health condition	−0.67 (1.04)	0.52
Autism symptoms	−0.17 (0.08)	0.03
Adaptive skills	0.09 (0.08)	0.29
*Stress 6 months*		
Stress baseline	0.57 (0.07)	<0.001
SS baseline	−0.15 (0.05)	0.006
Education	1.76 (0.72)	0.02
Household income	−0.44 (0.19)	0.02
Child health condition	−0.01 (1.14)	0.99
Autism symptoms	−0.10 (0.09)	0.31
Adaptive skills	−0.06 (0.08)	0.48
*SS 12 months*		
SS 6 months	0.46 (0.08)	<0.001
SS baseline	0.44 (0.07)	<0.001
Stress 6 months	−0.05 (0.06)	0.36
*Stress 12 months*		
Stress 6 months	0.33 (0.09)	<0.001
Stress baseline	0.41 (0.08)	<0.001
SS 6 months	−0.01 (0.04)	0.77

*SS = perceived social support*.

The cross-lagged path from baseline social support to stress at 6 months was significant (*b* = −0.15, *p* = 0.006), indicating that higher baseline social support is associated with lower levels of stress at 6 months. All other cross-lagged paths were non-significant.

## Discussion

Results shed light on how perceived support uniquely relates to parent stress and child problem behavior. As expected, there was remarkable stability in our three variables over a 1-year period. In community samples, it is common to find that without major treatment initiatives, many of the stressors associated with chronic neurodevelopmental conditions are themselves chronic, adding to the level of short term relative stability in the presence of behavior problems, which may take longer to change than a 1-year period (52). Stress itself has been described as a state that is associated with stable personality traits, such as neuroticism (53), it is likely that these parent characteristics also inserted a degree of relative consistency across respondents. Given that we can expect little change in stressors, stress, and support within a year, it is important to consider ensuring that families have access to interventions to address their needs in a timely manner to foster greater positive change.

Coupled with the fact that this stability was confirmed over a 1-year period, and that we controlled for many additional potential confounding variables, including parent education level and family household income, child health conditions, and the level of children's adaptive and autism symptoms, it is perhaps unsurprising that the degree of cross-lagged variance accounted for in our models was small though significant in the first 6-month period. Cross-lagged models are unique in that they control for variable stability across time points and are better equipped to assess reciprocal relationships. This methodology has been used in the autism research field to elucidate the reciprocal relationships among expressed emotion and behavior problems in adults (54), child anxiety and over-responsivity (49), adolescent behavioral development and vocational engagement (35), and child behavior and parent well-being (38, 55). In the current analysis, baseline perceived social support predicted 6-month child behavior and 6-month stress, but neither of the latter variables predicted subsequent social support. Greater within-subject variability over time may have resulted in more observations of cross-lagged effects.

The existing research framing child behavior problems as a determinant of perceived social support posits that caregivers may struggle to mobilize supports or are more reluctant to seek support when their children have more difficult behaviors [e.g., (3)]. This pattern was evident in our bivariate correlation analyses, as perceived social support was negatively associated with increased child behavior problems. However, results did not confirm this pattern longitudinally. Specifically, baseline perceived support significantly predicted subsequent child behavior problems at 6 months such that higher levels of perceived social support led to lower levels of child behavior problems, but child behavior did not predict subsequent social support. Research is scant on the potential mechanisms leading from social support to child behavior in the general population. One explanation is that perceived social support influences parenting practices which, in turn, affects child behavior. For instance, Hashima and Amato (56) found that perceived support was negatively associated with punitive parenting practices. Correspondingly, higher levels of parent social support have been associated with increased child praising and less controlling parent behavior (57). Increased social support and a rich social network may expose parents to positive practices or reinforce parenting norms through social pressure (58). The association between parenting practices and perceived support was noted in one study involving parents of children with autism, where perceived social support was correlated with increased perceived limit setting ability, maternal involvement, and satisfaction with parenting (59).

Baseline support was also found to lead to decreased stress at 6 months, though the path from 6-month support to 12-month stress was not significant. This result provides partial support for the hypothesis that social support is a resource that may alleviate parent stress, even when past stress levels and known stressors are controlled (e.g., education level, income, child autism symptoms, adaptive skills). There was no evidence that higher stress levels lead to perceived support. These results are consistent with the single existing study examining this bidirectional relationship longitudinally for mothers of children without neurodevelopmental conditions. Green and Rodgers (60) reported that baseline perceived social support predicted perceived stress 1 year later, but stress did not predict subsequent social support over and above baseline social support. Further, in a longitudinal study involving 283 Canadian mothers of young children with autism, higher perceived social support at baseline was associated with lower levels of subsequent parent stress 2 years later (61), but the opposite effect was not investigated. These findings are consistent with cross-sectional studies.

### Limitations

This study has a number of limitations. Participants were recruited through community organizations and a research lab database, and thus parents were likely engaged with autism services or had previously been active in research activities. Parents were mainly well-educated mothers living in suburban or urban locations and nearly all children were born in Canada. Further work with more diverse samples and comprehensive national recruitment strategies is needed as the current study results may not generalize to all parents of children with autism. Second, the data were collected through self-report surveys and it is possible associations among variables are inflated due to shared method variance. We relied on parent report of the autism diagnosis source (e.g., pediatrician, psychologist), diagnosis date, and parent report SCQ scores. Although the SCQ has been found to a valid screener for autism symptoms, in-person diagnostic testing is ideal. Additionally, the current study investigated social support over a 12-month period and future research should study social support over longer periods of time to better understand patterns of change. Furthermore, survey measures used different time periods of reference and this may have influenced the strength of associations. For instance, the measure of stress asked participants to consider the previous week, while the received support measure focused on the previous 4 weeks. Adjusting the time point reference for consistency would be something to consider for future studies. Finally, future studies could examine other dimensions of social support (e.g., social network characteristics, support needs support from specific sources), assess stress within specific contexts (e.g., parenting stress), or consider other social support determinants such as date of autism diagnosis (61), familial interactions from early childhood (30), parenting practices [e.g., (62)], and personal predispositions [e.g., (63)].

### Conclusions

After controlling from socioeconomic status, health status and autism symptomology, the stability of perceived social support, parental stress and child behavior challenges were clearly demonstrated. Consistent small to moderate concurrent relationships between perceived social support were and parent stress were found, with minimal evidence for cross-lagged relationships (perceived social support was related to parent stress and behavior problems 6 months later). Examining the concurrent and cross-lagged relationships between perceived social support and child behavior problems, no relationships were found between the 6- and 12-month time point. Given this stability, it is critical that interventions aim to address child behavior problems, stress, and also ways of shifting social support. To our knowledge, within the context of children with autism, there is no existing evidence-based intervention specifically targeting parents' perceptions of their support, though some multi-component programs have incorporated discussions on accessing social support (64–66). Given the presence of both considerable evidence for parenting stress programs (67) and programs to address child behavior problems (68), early access to methods of improving perceptions of social support would be a logical next line of intervention research.

## Data Availability Statement

The datasets presented in this article are not readily available because we are unable to provide access to the dataset related to this work for privacy and consent reasons. Requests to access the datasets should be directed to jonweiss@yorku.ca.

## Ethics Statement

The studies involving human participants were reviewed and approved by Human Participation Review Committee, York University. The patients/participants provided their written informed consent to participate in this study.

## Author Contributions

JW was responsible for overall supervision of the project, conceptualization, funding, and final manuscript preparation. SR was responsible for conceptualization, data collection and analysis, and initial manuscript preparation. RP and DF provided guidance for research design, data analysis, conceptualization, and contributed to manuscript preparation. All authors contributed to the article and approved the submitted version.

## Conflict of Interest

The authors declare that the research was conducted in the absence of any commercial or financial relationships that could be construed as a potential conflict of interest.
